# 

**Published:** 1896-01-18

**Authors:** 


**Notices of Births, Deaths, and Marriages** are inserted in this column at a charge of 2s. 6d. for an announcement not exceeding four lines.

**Notice to Advertisers and Subscribers.**

All Advertisements, Orders for Copies of The Hospital and Business Communications should be addressed to **THE MANAGER, (Not to The Editor),** The Hospital, 428, Strand, London, W.C.

The Cost of Subscription, post free, is as follows:—Per week, 2½d.; per quarter, 2s. 8d.; per half-year, 5s. 3d.; per annum, 10s. 6d. Foreign — Per Annum, 15s. 2d.; payable, in all cases, in advance.

**NOTICE TO CORRESPONDENTS.**

All MS., Letters, Books for Review, and other matters intended for the Editor should be addressed The Editor, The Lodge, Porchester Square, London, W.

The Editor cannot undertake to return rejected MS., even when accompanied by stamped directed envelope.

**"Burdett's Hospital Annual," 1895,**

Sixth year of publication. 950 pp. Scarlet cloth, gilt lettered. Price 5s. A most complete guide to British, Colonial, Indian, and American Hospitals, Dispensaries, Nursing and Convalescent Institutions, and Asylums. A few complete sets of the preceding five issues of the "Annual" may still be obtained on application to the Publishers, The Scientific Press (Limited), 428, Strand, London, W.C.

**NOTICE.—Vol. XVIII. of "The Hospital" (April to September, 1895), in handsome cloth binding, is on sale at the Office of this Journal. Price 7s. 6d. Cases for binding the Numbers contained in this Volume, 1s. 6d. Index to Vol. XVIII., 2½d., post free. The Monthly Part for January is now ready, price 6d., by post, 8d.**

### SCRAPS AND GLEANINGS.

THE Queen has sent a present of game to St. George's Hospital for the use of the patients.

LADY GREGORY (Mrs. Sterling) has bequeathed £250 free of legacy duty to the London Ophthalmic Hospital.

THE Prefect of Constantinople has ordered a complete inspection to be made at Constantinople, with a view to effecting a complete sanitary reform in the city.

A SUM of £1,929 has been paid as a legacy to the Edinburgh Royal Infirmary and Convalescent House by the executors of the late Dr. A. Reid, of Hankow, China.

So large a percentage of foreign doctors are practising in Paris that the Chamber of Deputies and the Senate are considering what measures to enforce with a view to checking this influx.

Some weeks back public circulation was given to a statement that a fire had occurred at the Sheffield General Infirmary. We are requested to contradict this erroneous information, no such fire having occurred at the premises.

A SERIOUS fire broke out the other day in the Hospital of the Mullingar District Lunatic Asylum, at a time when 40 patients were in bed, some of them being carried on mattresses out of danger. A portion of the building was entirely destroyed, but no lives were lost.

THE in-patients during last year at the Hospital for Diseases of the Throat, Golden Square, numbered 610. Subscriptions to the Building Fund, which is to take the form of a memorial to the late Sir Morel Mackenzie are not flowing in as fast as could be desired. Two legacies, however, of £1,000 each have enabled the Building Fund Committee to undertake the much-needed addition.

FOR many years past the work at the Sheffield General Infirmary has been greatly retarded by the want of necessary accommodation for the sisters and nurses and of special wards for the treatment of diseases of the eye and skin. A special appeal is now being made for £20,000 for these necessary alterations, and it is hoped this sum will shortly be forthcoming, for the work of the infirmary is spread over a wide area.

THE committee of the British Asylum for Deaf and Dumb Females are appealing for help to support them in their work among deaf mutes. The charity has existed now for upwards of forty-two years, and is, at the present moment, in great need of funds to meet the yearly increasing claims upon it. The asylum is situated at Lower Clapton, E., and is under the patronage of T.R.H. the Prince and Princess of Wales.

THE twenty-second annual meeting of the governors of the Holmesdale Cottage Hospital, Maidstone, has lately been held. During the period under review 65 patients have been under treatment in the hospital, and the work of the institution has been carried on quietly and efficiently. Though the subscription list shows a slight increase this year, it has not been quite up to the standard of previous years. A legacy of £100 has been bequeathed to the hospital.

AMONG the many societies which appeal to us the Royal Society for the Prevention of Cruelty to Animals deserves much consideration at our hands. The work this society does in the cause of our dumb friends is gradually extending all over the world. To carry on this work, there is need for much help with its funds. Every year about 7,500 convictions are registered, and upwards of 200 branches and auxiliaries are now added to the parent branch, the offices of which are at 105, Jermyn Street, St. James.

BISHOP BLYTH is doing work of a two-fold character in the East. On the one hand his labours are among the English people at Suez, Port Said, Beyrout, etc.; and on the other hand he is engaged in mission work among the Jews at Jerusalem, and elsewhere in the East. Generous help is required for the hospital at Haifa, where a fully-qualified doctor is in charge, assisted by three trained ladies. Subscriptions to the Jerusalem and the East Mission may be addressed to the Rev. C. Berkeley, Belton Vicarage, Uppingham.

### BOOKS RECEIVED.

HORNER AND SON, WHITBY.
"Health Notes for the Seaside, with special reference to Whitby and District." By A. C. Dutt, B.A., M.B.

LIQUOR CARNIS CO.

"Our Treasures and How to Keep Them."

SIMPKIN, MARSHALL, AND COMPANY.
"Gout and Rheumatic Gout." By Dr. Alfred Wright.

J. AND A. CHURCHILL.
"History of the Cholera Controversy." By Sir George Johnson, M.D.

**Periodicals and Pamphlets.**—Literary Digest and Medical Press, Annals of Surgery, International Medical Magazine, The Zenana, Dagny, Medical Week, British and Colonial Druggist, Indian Medico-Chirurgical Review, Clinical Journal, Leeds Hospital Magazine, New York Medical Journal, Nursing Notes, Hunter's Weekly, London, Endeavour Herald, Invention. Guy's Hospital Gazette, Fortieth Annual Report of the National Sanatorium for Consumption and Diseases of the Chest, Annual Report of Tower Hamlets Pension Committee, Popular Science, Practitioner, Humanitarian, Hope, Charlotte Medical Journal, Westminster Review, Monthly Homœopathic Review.

### THE STUDENT OF MEDICINE.

### APPOINTMENTS.

Dr. Francis, reappointed Medical Officer for the Markeaton District of the Belper Union. Dr. N. T. Hannah, appointed Medical Officer for the Buxton District of the Chapel-en-le-Frith Union. G. R. Harcourt, M.R.C.S., L.R.C.P.Lond., appointed Assistant Medical Officer and Dispenser at the Lambeth Infirmary. R. G. McKerron, M.A., M.B., appointed Junior Physician to the Royal Aberdeen Hospital for Sick Children. H. Menzies, M.B., B.C.Camb., appointed Obstetric Assistant to St. George's Hospital. Mr. A. Morrison, appointed Assistant Medical Officer to the St. Pancras Infirmary. E. S. Nutting, M.B., C.M.Edin., appointed Medical Officer for the No. 3 District of the Mansfield Union. H. H. L. Patch, M.R.C.S., L.R.C.P.Lond., appointed Medical Officer for the Chudleigh District of the Newton Abbot Union. G. A. Simmons, M.D., B.S Lond., M.R.C.S.Eng., L.R.C.P.Lond., appointed Medical Officer to Fairfield House, Tooting. L. M. Snow, appointed Medical Officer and Public Vaccinator for the No. 2 District of the Horsham Union. J. T. Thomas, L.R.C.P.Lond., M.R.C.S., appointed Medical Officer of Health to the Camborne District

### THE STUDENT OF MEDICINE—*continued.*

Council. W. Turner, M.B., B.S.Lond., F.R.C.S.Eng., appointed Sambrooke Surgical Registrar to King's College Hospital. W. F. W. Wilding, M.R.C.S., L.R.C.P., appointed Surgeon for Hindley District of the Wigan Junction Colliery. Dr. R. Wilson, appointed Medical Officer for the Newchurch District of the Haslingden Union. T. B. Young, M.D.Brux., M.R.C.S.Eng., L.R.C.P., appointed Medical Officer of Health to the Halesowen Urban District. Miss R. C. Despard, M.B.Lond., has been appointed a Junior Assistant Medical Officer at the Holloway Sanatorium Hospital for the Insane, Virginia Water.

### VACANCIES.

The following vacancies are announced this week, the amount of salary in each case being given after the name of the institution:—

#### Resident Medical Officers.

Birmingham General Dispensary.—Resident Surgeon; doubly qualified. Salary £150 per annum, with allowance of £30 per annum for cab hire, and furnished rooms, fire, lights, and attendance. Applications to Alex. Forrest, Secretary, by January 20th.

County Borough of Cardiff.—Resident Medical Officer for the Borough Hospital for Infectious Diseases; unmarried. Appointment for one year. Salary £50 per annum, with board (without stimulants) and residence in the hospital. Applications to Dr. Walford, Medical Officer of Health, Town Hall, Cardiff, by January 18th.

North Staffordshire Infirmary and Eye Hospital, Hartshill, Stoke-upon-Trent.—Assistant House Surgeon. Board, washing, and apartments provided. Applications to the Secretary by January 27th.

Scottish Prison Service.—Resident Medical Officer. Salary £250, with a house. Applications to the Secretary of the Prison Commission for Scotland, 6, Rutland Square, Edinburgh, by January 31st.

#### Non-Resident Medical Officers.

Children's Hospital, Nottingham.—House Surgeon. Appointment for six months, but eligible for re-election. Salary at the rate of £100 per annum. Applications to the Secretary by January 20th.

London Hospital.—Assistant Anæsthetists; must be registered members of the profession. Appointments for six months, but eligible for re election. Salary at the rate of £50 a year will be paid. Applications to Monro Scott, Warden, by January 18th.

Royal Free Hospital, Gray's Inn Road.—Assistant Physician to out-patients; must be F. or M.R.C.P.Lond. Applications to the Secretary by January 25th.

University of London.—Registrarship. Salary commences at £800, and rising by annual increments to £1,000 per annum. Applications to Arthur Milman, M.A., LL.D., Registrar, University of London, Burlington Gardens, W., by January 25th.

### UNIVERSITIES AND COLLEGES.

#### Royal College of Surgeons of Edinburgh.

The following gentlemen, having passed the requisite examination, have been elected Fellows of the College: L. W. Bickle, L.R.C.P. Lond., and M.R.C.S. Eng., Mount Barker, South Australia; E. G. Fortune, M.B., C.M., Edin., Demonstrator of Surgery, University of Edinburgh, 20, Chalmers Street, Edinburgh; A. W. Hall, L.R.C.P. and S. Edin., and L.F.P. and S. Glasg., Scopwick, near Lincoln; F. M. Graham, L.R.C.P. and S. Edin., and L.F.P. and S. Glasg., resident medical officer, Cowgate Dispensary, Edinburgh; J. L. Yuill, M.B., C.M., Glasg., Burnbank Road, Hamilton; T. H. Morgan, M.B., C.M., Edin., consulting surgeon, Glympie Hospital, Queensland; T. B. Kelly, L.R.C.P. and S. Edin., house surgeon, Eye Hospital, Lower Mandlin Street, Bristol; W. J. C. Nourse, L.R.C.P. and S. Edin., and L.S.A. Lond., honorary surgeon, Holloway and North Islington Dispensary, 28, Parkhurst Road, London, N.; A. S. Ferguson, M.R.C.S. Eng. and L.R.C.P. Lond., 1, Lansdowne Place, Guildford Street, London, W.C.; F. T. Anderson, L.R.C.P. and S. Edin., 20, Inverleith Row, Edinburgh; A. K. Melville, L.R.C.P. and S. Edin., M.D. and C.M. Edin., 35, Mayfield Gardens, Edinburgh; G. C. A. Moir, M.R.C.S. Eng., and L.R.C.P. Lond., assistant surgeon, St. Paul's Eye and Ear Hospital, 35, Merton Road, Bootle, Liverpool; W. A. Bonney, M.R.C.S. Eng., L.S.A. Lond., L.R.C.P. Edin., M.D. Brussels, 100, Elm Park Gardens, London, S.W. The Bathgate memorial prize, awarded annually after a special written examination in materia medica and therapeutics, and open to all students who have taken their course of materia medica at the School of Medicine of the Royal Colleges, and who have passed in the second examination under the old regulations or the third examination under the new regulations of the triple qualification, was, at a meeting of the College held yesterday, presented to the successful candidate, Mr. Patrick Pearse, 1, Argyle Place.

#### University of London.

B.S. Examination for Honours in Surgery.

First Class.—*W. Turner, King's College; †A. E. Russell, St. Thomas's Hospital; ‡H. B. Shaw, University College; Charlotte Elizabeth Hull, Royal Free Hospital; G. B. Hunt, University College; J. S. Sloane, B.Sc., St. Bartholomew's Hospital; W. T. G. Pugh, Middlesex Hospital; H. L. Barnard, London Hospital.

Second Class.—T. M. Thomas, Guy's Hospital; Frances May D. Berry, M.D., London School of Medicine and Royal Free Hospital; D. A. Chaning-Pearce, Guy's Hospital.

* Scholarship and Gold Medal. † Gold Medal. ‡ Obtained the number of marks qualifying for a Gold Medal.

#### University of Dublin.

At the winter commencements, held on Friday, December 20th, 1895, the following degrees and licences in Medicine were conferred by the Unive sity Caput in the presence of the Senate, assembled in the Theatre of Trinity College:

Licentiatus in Medicina, in Chirurgia, et in Arte Obstetricia.—A. J. Somers.

Baccalaurei in Medicina, in Chirurgia, et in Arte Obstetricia.—J. Beatty, B.Ch. (stip. cond.), P. P. Carton, W. J. Dawson, P. N. Gerrard, H. R. Harley, E. Hemphill, F. S. T. Hutchison, H. E. Littledale, L. F. M'Dowell, R. C. Peacocke, H. R. Robertson, W. N. Walker, J. Winder, F. A. Winder.

Doctores in Medicina.—W. Ayres, G. E. Crowe, L. W. Crowe, T. Edwards, A. R. Friel, W. W. Grosvenor, H. R. Harley, A. H. Holmes, R. W. W. Henry, C. A. Johns, G. W. Kendall, E. H. Montgomery, A. A. Mussen, H. R. Robertson, A. H. S. Todd, E. H. Townsend.

In Absentia.

Doctore in Medicina.—G. A. Wade.

### Medical and Administrative Appointments.

GREAT NORTHERN CENTRAL HOSPITAL, Holloway, N.

Notice is hereby Given, that a VACANCY will occur on 7th February next for a JUNIOR HOUSE SURGEON. Board, Lodging, and Laundry provided in the Hospital.

Candidates, who must possess a registrable qualification, are requested to send in their application, together with 36 copies of their testimonials, on or before MONDAY, the 27th January inst.

Forms of application and full particulars may be obtained from the undersigned.

LEWIS H. GLENTON KERR, Secretary.

January 8th, 1896. (339)

DUNDEE ROYAL LUNATIC ASYLUM.

RESIDENT CLINICAL ASSISTANT. No Salary, but Board and Residence.

Apply to Dr. Rorie, Westgreen House, Dundee. (385)

Demy 8vo., with Two Plates, handsome cloth gilt, over 400 pp., price 12s. 6d. net.

MEDICAL HISTORY FROM THE EARLIEST TIMES.

By E. T. WITHINGTON, M.A., M.B.Oxon.

The Scientific Press (Limited), 428, Strand, London, W.C.

### "THE HOSPITAL"

AN INDEPENDENT JOURNAL OF
**The Medical Sciences**
AND
**Hospital Administration,**
WITH WHICH IS ISSUED WEEKLY
**A SPECIAL**
### SUPPLEMENT FOR NURSES.

PUBLISHED EVERY SATURDAY.
PRICE TWOPENCE.

**Matrons, Sisters, or Nurses will find in The Hospital a chronicle of the latest Nursing News, Practical Articles of the highest value to those who wish to keep themselves abreast with the times, and brightly written articles from Nurses all over the world.**

**SUBSCRIPTIONS CAN COMMENCE AT ANY TIME.**

### TERMS OF SUBSCRIPTION.
### WEEKLY ISSUE.

| | Great Britain. | Foreign and Colonial. |
|---|---|---|
| For One Year ... ... | 10s. 6d. ... | 15s. 2d. |
| For Six Months ... | 5s. 3d. ... | 7s. 7d. |
| For Three Months... | 2s. 8d. ... | 3s. 10d. |

### MONTHLY PARTS.

| | Post Free to any Part of the World. |
|---|---|
| For One Year ... ... | 8s. 6d. |
| For Six Months ... ... | 4s. 3d. |
| For Three Months ... ... | 2s. 2d. |

Postal Orders and Cheques should be made payable to "The Scientific Press, Limited."

N.B.—In order to prevent delay, all business communications should be addressed to "The Manager" only, and not to any person individually.

### CHANGE OF ADDRESS.

In event of change of address, notice should be forwarded to the Publishers by the Saturday previous to date of publication, and the old address should also be given.

"The Hospital" can be obtained of all Booksellers and Newsagents in the United Kingdom, and at all the Railway Stalls, of Messrs. W. H. Smith and Son; and also from the following Agents in the Colonies and Abroad:—

Paris: Librairie Galignani, 224, Rue de Rivoli.
Berlin: Messrs. A. Asher and Co.
Leipsig: Mr. F. A. Brockhaus.
New York: The International News Co.
Montreal: The Montreal News Co.
Toronto: The Toronto News Co.
Melbourne, Sydney, Adelaide, and New Zealand: Messrs. Gordon and Gotch.
India: At all the Railway Bookstalls and Agencies of Messrs. A. H. Wheeler and Co.
South Africa: Messrs. Juta, Cape Town.

Publishing Offices: 428, Strand, London, W.C.

*In the Press. Price 5s.*

### BURDETT'S OFFICIAL NURSING DIRECTORY

A DIRECTORY, NOT A REGISTER.

THE HOSPITAL states: "It is well to bear in mind the difference and distinction which exists between a register and a directory. A register implies rights, and suggests that those who are not upon it are in some way or another inferior beings. But a directory is an other matter. A directory gives as rights; insertion in it implies no adherence to any particular party or any particular opinion, and in no way interferes with entry on the register of any association, or the list of any institute, to which the individual may belong. All that a directory professes to do is to give information. The 'Medical Register' is not a mere list of qualified medical men; it is admission to the Register, which itself makes him qualified. For years, perhaps even now, 'Churchill's Medical Directory' contained the names of medical men who, so far as public appointments were concerned, were unqualified; it also contains the names of homœopathists, whom four out of every five medical men will not meet; and it certainly contains the names of many for whose moral character we should not like to vouch. But it gives full and accurate information about them all, and for the sake of that information it is constantly consulted both by the public and the doctors."

WHAT NURSES NEED.

"At present there is no Register for the nursing profession giving any legal status. Such a thing may come or it may not; but in either case there is no reason why there should not be a Directory, giving such information about the career of each nurse as both the public and the profession want; information, by the by, which no legal Register would be likely to insert. The 'Medical Register' is a bald and meagre thing compared with the 'Medical Directory.' What Burdett's 'Official Nursing Directory' proposes to do is to supply the want of a directory giving information. It will give no rights, it will imply no partisanship, it will give no guarantee except that what it prints is true, and is derived from official sources."

ACCEPTABLE TO ALL NURSES.

"This ought to be enough to make it acceptable to the public, and every nurse ought to make sure that, whatever list or register she may be on, her name shall also appear on Burdett's 'Official Directory.'" Applications for forms should be made at once to the Editor, "Burdett's Official Nursing Directory," 428, Strand, W.C.

**The above work will be ready early in the Spring, and those Nurses who have not already sent in their names should at once apply for a form, which can be obtained upon application to the Publishers,**

THE SCIENTIFIC PRESS (Lim.), 428, Strand, London, W.C.

London: THE SCIENTIFIC PRESS (Limited), 428, Strand, W.C.

Now Ready. Third and Revised Edition (twenty-fifth thousand), demy 16mo. (suitable for the apron pocket), handsomely bound in cloth boards, 160 pp., price 2s. In handsome leather, gilt, price 2s. 6d., net.

### THE NURSE'S DICTIONARY
### OF MEDICAL TERMS AND NURSING TREATMENT.

**Compiled for the use of Nurses, by HONNOR MORTEN.**

Containing descriptions of the Principal Medical and Nursing Terms and Abbreviations, Instruments, Drugs, Diseases, Accidents, Treatments, Physiological Names, Operations, Foods, Appliances, &c., &c., encountered in the Ward or Sick-room.

**NURSING BOOKS AT FULL DISCOUNT.**

*LIST POST FREE.*

The Theory and Practice of Nursing (Percy Lewis, M.D.), 3/6 for **2/8.**
The Nurse's Dictionary (Honnor Morten), 2/- for **1/6.**
The Nurse's Case Book, 6d. for **3½d.**

